# A Retrospective Evaluation and Review of Radiographic Outcomes for Anterior Cervical Discectomy and Fusion (ACDF) Procedures: Northern Ireland's Experience

**DOI:** 10.7759/cureus.38864

**Published:** 2023-05-11

**Authors:** Christopher McKee, Robert Espey, Amanda O'Halloran, Adrian Curran, Nagy Darwish

**Affiliations:** 1 Medicine, Dentistry and Biomedical Sciences, Queen's University Belfast, Belfast, GBR; 2 Orthopaedic Surgery, Belfast Health and Social Care Trust, Belfast, GBR; 3 Orthopaedic Surgery, Royal College of Surgeons in Ireland, Dublin, IRL

**Keywords:** cervical disc degeneration, acdf, zero-profile, stand-alone, anterior cervical discectomy and fusion

## Abstract

Introduction

Anterior Cervical Discectomy and Fusion (ACDF) is the gold standard treatment for symptomatic cervical spondylosis refractory to analgesic medical management. Currently, there are numerous approaches and devices used; however, there is no single implant that is preferred for this procedure. The aim of this study is to evaluate the radiological outcomes of ACDF procedures performed in the regional spinal surgery centre in Northern Ireland. The results of this study will aid surgical decision-making, specifically with regard to the choice of implant. The implants that will be assessed in this study are the stand-alone polyetheretherketone (PEEK) cage (Cage) and the Zero-profile augmented screw implant (Z-P).

Methods

A total of 420 ACDF cases were reviewed retrospectively. Following exclusion and inclusion criteria, 233 cases were reviewed. In the Z-P group, there were 117 patients, with 116 in the Cage group. Radiographic assessment was carried out at the pre-operative stage, day one post-operation, and follow-up (> three months). Measured parameters included segmental disc height, segmental Cobb angle, and spondylolisthesis displacement distance.

Results

Patient characteristic features showed no significant difference between the two groups (p>0.05) and no significant difference in mean follow-up time (p=0.146). The Z-P implant was superior in increasing and maintaining disc height post-operatively (+0.4±0.94mm, 5.20±0.66mm) compared to the Cage (+0.1±1.00mm, 4.40±0.95mm) (p<0.001). Z-P was also more successful in restoring and maintaining cervical lordosis in comparison to the Cage group, as it had a significantly smaller kyphosis incidence (0.85% vs. 34.5%) at follow-up (p<0.001).

Conclusions

Results of this study show a more advantageous outcome in the Zero-profile group as it restores and maintains both disc height and cervical lordosis; it is also more successful in treating spondylolisthesis. This study advocates cautious endorsement of the use of the Zero-profile implant in ACDF procedures for symptomatic cervical disc disease.

## Introduction

Anterior cervical decompression and fusion (ACDF) is a widely recognised surgical option used to treat cervical spondylosis. Its basis is to restore normal anatomical structure by achieving increased disc height and stability of the vertebral column ​[1]. The standalone cage (Cage) was developed for this purpose, with the polyetheretherketone (PEEK) cage providing similar rigidity to normal bone. Numerous studies have since suggested that the use of the stand-alone cage alone results in segmental kyphosis and loss of disc height. It has been hypothesised that the progressive vertebral body wedging observed in patients treated with this construct causes subsequent kyphosis ​[2-4]​. Multiple centres perform anterior plate fixation in addition to Cage implantation to prevent these complications. Despite providing superior bone fusion and cervical alignment, anterior plating is not without complications. Due to possible soft tissue irritation, dysphagia is relatively common in patients with anterior plating compared to those treated solely with the Cage [3]. This has prompted the development of alternative devices, one such example being the Zero-profile augmented screw implants (Z-P) ​[5]​. Evidence has demonstrated the benefit of using Z-P in comparison to conventional anterior plating due to the fixed angle augmented screws providing a more rigid construct ​[6-8]. One previous study directly compared the radiographic outcomes of the Cage implant with those of a Z-P device in patients without anterior plating, demonstrating positive results for both implants. At follow-up, the latter device had a statistically significant superior outcome [9].

In Northern Ireland, both implants are routinely used for all ACDF procedures at the high-volume regional spinal surgery unit. Both implants are packed with demineralised bone matrix (DBM) to accelerate and ensure bone fusion. Due to the increased prevalence of dysphagia observed in anterior plating patients, this procedure is not performed at our centre for radiculopathic or myelopathic patients with cervical degenerative disc disease. The aim of this study is to evaluate radiographic outcomes in patients treated for cervical spondylosis through ACDF in Northern Ireland over the last five years. The results of this study will provide valuable insight into the biomechanics of the Cage and Z-P devices and help influence the choice of the most clinically advantageous implant for patients with cervical disc disease. This will help reduce post-operative complications and minimise the overall revision rate.

## Materials and methods

Patients’ selection 

A total of 420 patients underwent 530 ACDF procedures for cervical spondylosis between April 2015 and December 2021. All procedures were performed on patients presenting with radiculopathy or myelopathy at the regional spinal surgery centre. Each operation was performed by a single surgeon, with a total of eight spinal orthopaedic surgeons across all cases. Retrospectively, all cases were reviewed, and their clinical and demographic information was recorded. Patients included in this study were: (I) treated using a Cage or Z-P device; (II) under 65 years of age at the date of operation; (III) date of operation within the last five years (April 2015-December 2021); (IV) ACDF performed at levels C3/4 to C7/T1. The exclusion criteria for this study were as follows: (I) trauma involved in the mechanism of injury; (II) pre-existing spinal deformity or disease (i.e., ankylosing conditions, scoliosis, metastases); (III) previous cervical spine surgical intervention; (IV) additional instrumentation (i.e., posterior stabilisation or anterior plating); (V) age at the date of operation greater than 65; (VI) follow-up period shorter than three months. After applying these criteria, a total of 233 patients were reviewed radiographically over a two-month period (June-July 2022).

Surgical technique 

**Figure 1:**
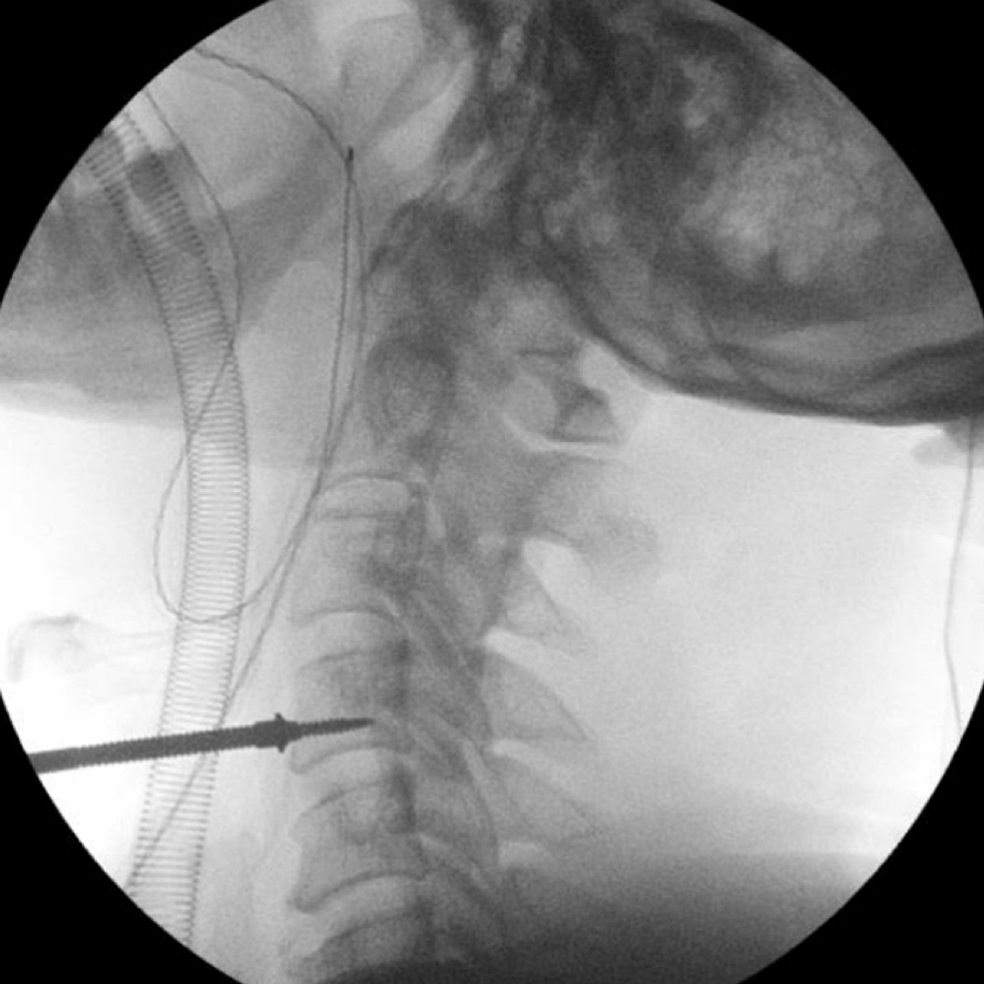
Intraoperative fluoroscopy image displaying confirmation of the C4 vertebral level with a 40 mm Caspar distracting pin. The operative level in this particular case is the C4/5 segment.

As per the records, an anterior Smith-Robinson approach was performed in all cases. The vertebral level was confirmed using C-arm fluoroscopy prior to disc intervention by the insertion of a Caspar distracting pin placed into the vertebral body above or below the level in question (Figure 1).

Vertebral body margins were prepared using a curette, and an additional distraction pin was inserted into the adjacent vertebral body into the diseased disc space. A Caspar cervical distractor was then used to restore natural disc height. The degenerative disc was removed using a combination of Kerrison rongeurs and pituitary forceps, decompressing the spinal cord or compressed nerve root. Osteophytes and remaining annular tissue were removed using a burr or Kerrison bone punch. The sizing of the implant was then performed using a template. The chosen implant was then inserted into the disc space, with its anterior border placed in line with the most anterior point of the vertebral bodies. In the Z-P group, locking screws were inserted under C-arm x-ray guidance at a 27°-44° angle in the cranial-caudal direction. Both Z-P and Cage devices were pre-packed with demineralised bone matrix (DBM) as per the standard procedure. After the insertion of each implant, intraoperative fluoroscopy images were taken to confirm cage positioning and ensure adequate alignment. The cages have titanium alloy markers anteriorly and posteriorly to aid location confirmation. The correct positioning of the Z-P spacer devices is indicated by all screws entering the middle of the vertebral body above and below. Haemostasis was performed when necessary, and incisions were closed with sutures by routine layer closure.

Data collection 

Patient demographic and clinical characteristics were provided by the Fracture Outcomes Research Department (FORD) within the Royal Victoria Hospital, Belfast, Northern Ireland. Demographic and clinical dependent variables included age, sex, diagnosis, and the American Society of Anaesthesiologists (ASA) score. Other clinical variables, including level of operation, procedure, and implant used, were obtained from the Belfast Orthopaedic Information System (BOIS). Two imaging systems were used to obtain images for radiographic analysis: Sectra PACS (Sectra Medical) and Intellispace Portal 9.0 (Koninklijke Philips Healthcare N.V.).

Radiographic analysis 

**Figure 2:**
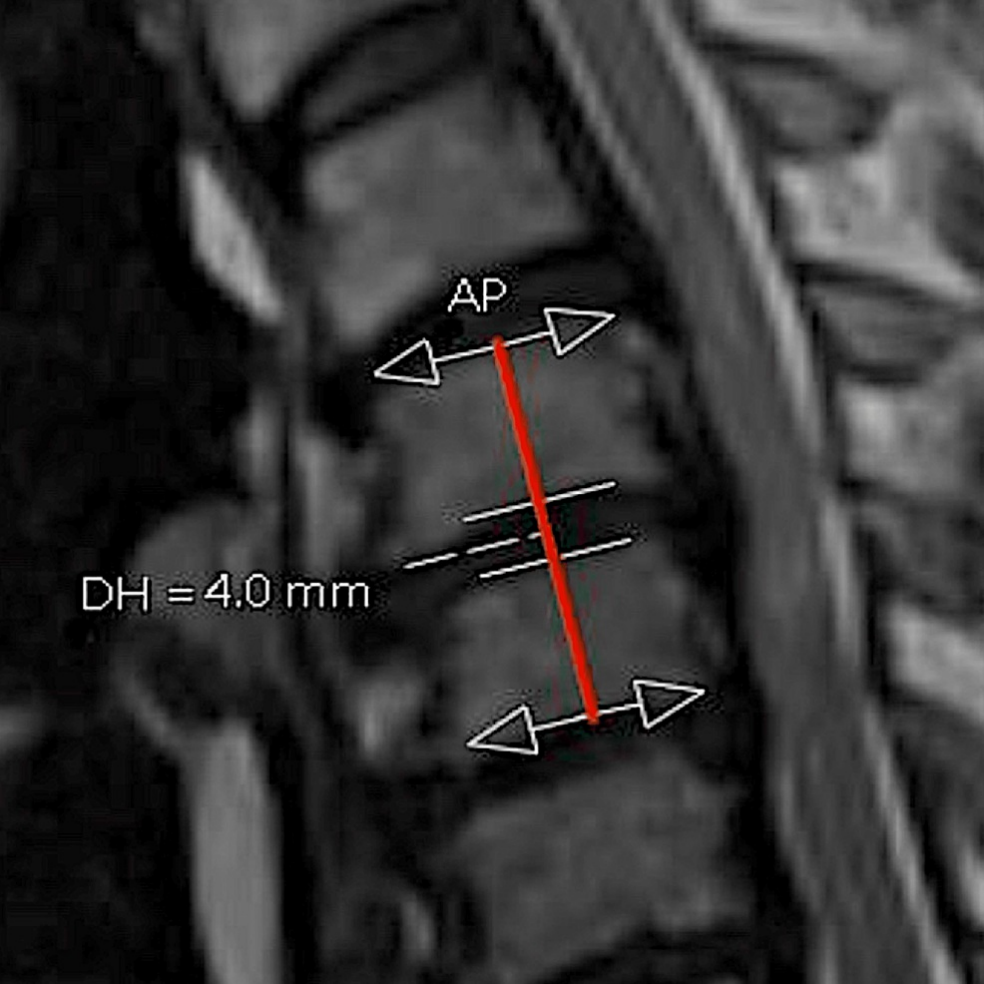
Sagittal T2 MRI of a patient with severe cervical stenosis at levels C3/4 to C6/7. The anteroposterior length (AP) of the end plate is indicated by the white arrow. Disc height (DH) is measured between the parallel white lines at the halfway point between AP (indicated by the red line). Measurements were taken between the superior and inferior endplates of the disc space. DH: disc height; AP: anteroposterior length

Patients were reviewed using two imaging modalities: routine pre-operative whole spine magnetic resonance imaging (MRI) and cervical plain film radiographs with anteroposterior and erect lateral views. All patients were reviewed at three separate time periods: pre-operative (pre), post-operative (PO), and follow-up (FU). A pre-operative MRI was performed within three months of surgery. The post-operative x-rays were taken on day one post-surgery, and the follow-up was after three months. At this regional spinal centre, patients are routinely reviewed at three months and only reviewed after this time if they are symptomatic, have post-operative complications, or present with other spinal pathology (e.g., lumbar degenerative disease). Numerous patients (44.6%) were further reviewed at six, 12 and 24 months, depending on disease severity. Vertebral disc height (DH) was measured at each time point to calculate segmental subsidence (Figure 2).

**Figure 3:**
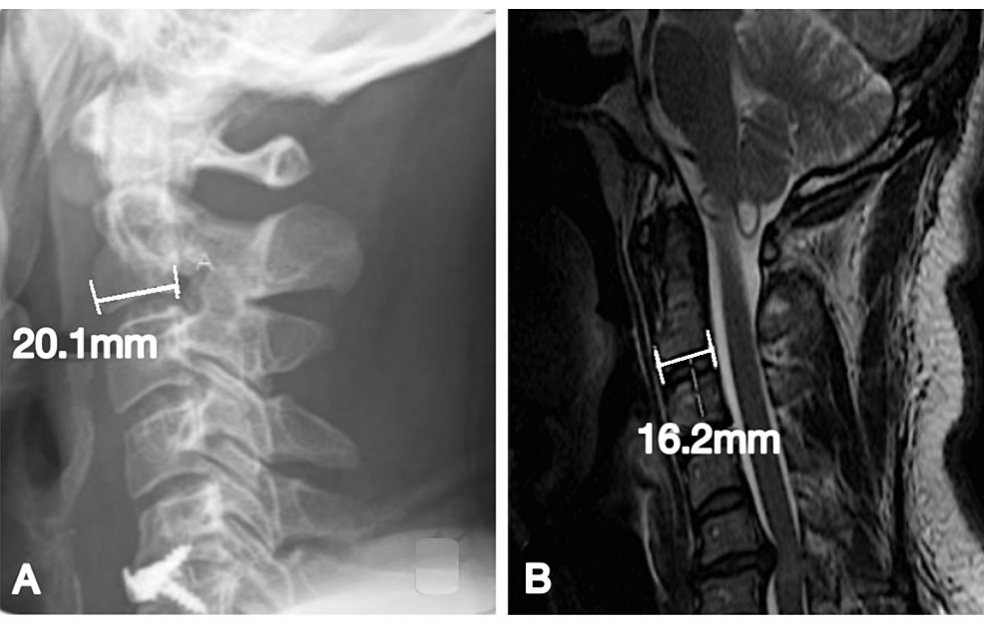
(A) Sagittal X-ray and (B) MRI of the same patient treated with Z-P. Measurement AP indicates the anteroposterior length of the inferior endplate of the C2 vertebrae in both figures. Note the difference in AP values between imaging modalities, highlighting the importance of the formula stated above. This prevents measurement errors in both imaging modalities.

Due to the magnification discrepancy between MRI and X-ray, the anterior-posterior length of the C2 vertebra was measured in all images to calculate actual disc height (aDH), as shown in Figure 3. The C2 vertebra was chosen as a landmark to calculate the ratio between MRI and X-ray magnification, as outlined by Shigematsu et al. [10]. The X-ray C2 length (AP) was calibrated in comparison to the length measured on MRI, as the latter demonstrates actual vertebral length (aAP). This ensured the accuracy and consistency of each disc's height measurement. The resulting formula was: aDH = aAP/AP x DH, calculated by the author of the current study.

**Figure 4:**
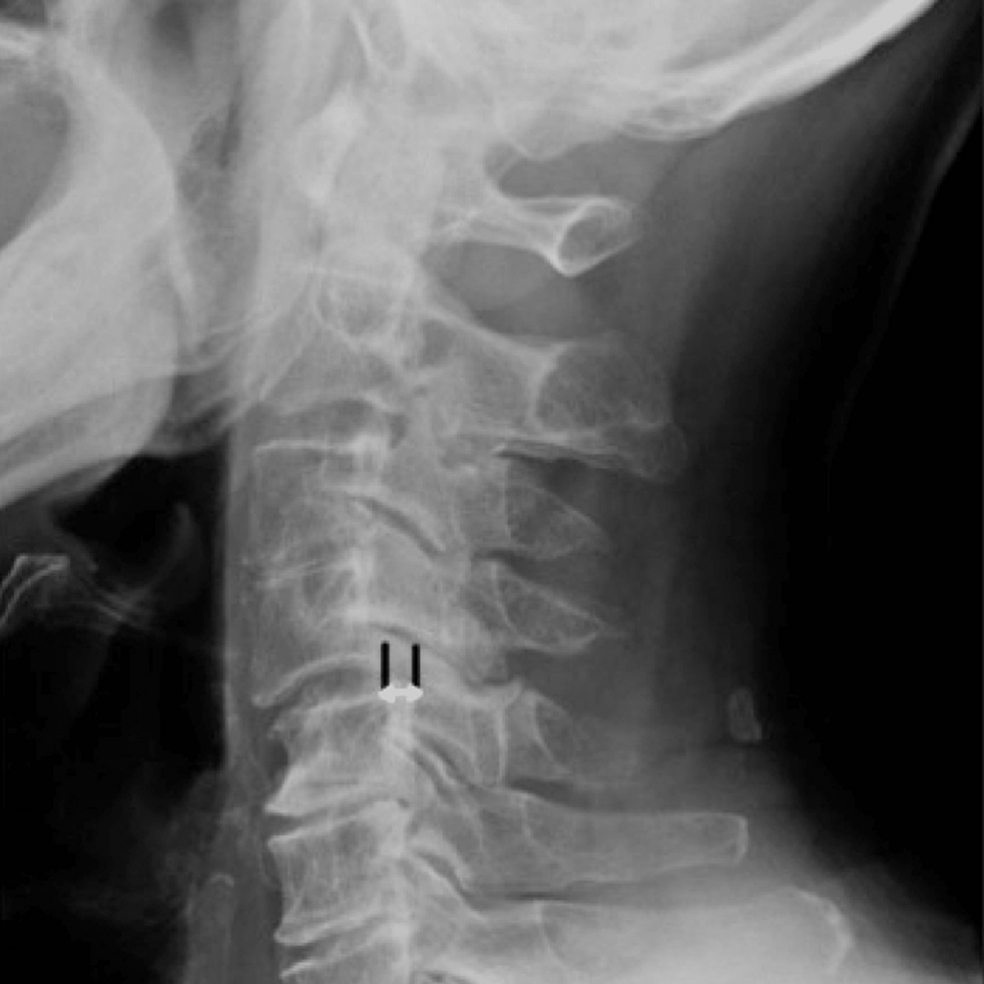
Lateral radiograph showing spondylolisthesis. The extent of spondylolisthesis was measured as the tangential distance between the two parallel lines, making the posterior corner of the cranial and caudal vertebrae of the affected level. Image adapted from [11]

Segmental Cobb angles (s-Cobb) were measured to evaluate the exacerbat2 mm.ion of kyphosis at each time point. A kyphosis angle was classified as a positive value, while a lordosis angle was assigned a negative value. No calibration was required for both baseline MRI and lateral X-ray angle measurements. Following a study conducted by Kim et al. [11], spondylolisthesis was measured as shown in Figure 4. Significant spondylolisthesis was considered to be of a value >2mm​. All cases with any degree of listhesis at the diseased vertebral level were measured. The aim was to determine if a significant number of this patient cohort suffered from spondylolisthesis in conjunction with their primary symptoms.

Inter-observer reliability

All images were reviewed by the author and two independent observers to measure inter-observer reliability and ensure measurements were accurate and consistent. Inter-observer reliability was determined for all three radiographic measurement types (aDH, s-Cobb, and spondylolisthesis). Two independent observers were given a random sample of 35 patients, generated by a random number generator. Each patient had three images to be reviewed, for a total of 105 images analysed by each independent observer. This was then compared to the measurement taken by the author to obtain an intraclass correlation coefficient (ICC) for each measurement. This method was used as all data contained continuous numerical values. An ICC >0.90 indicated excellent reliability, with 0.75-0.90 being good, 0.5-0.75 being moderate, and <0.5 being poor. These reliability scores are based on guidelines suggested by Koo and Li [12].

Statistical analysis 

Statistical analysis was performed using IBM Corp.'s Statistical Package for Social Sciences (SPSS 26.0) statistical software (SPSS Inc., Chicago, IL, USA). All values were expressed as the mean ± standard deviation. Quantitative data were analysed using the Mann-Whitney U test, while qualitative data were evaluated using the Chi-square test. Statistical significance was considered when the p-value < 0.05. Linear graphs were also generated using the above software.

Ethics 

Ethical approval or informed consent was not required for this study. The patient data acquired were kept anonymous in accordance with the Data Protection Act of 1998. Approval was provided by the Institutional Review Board within the Standards, Quality, and Audit Department at the Royal Victoria Hospital, Belfast, United Kingdom.

## Results

Patient demographics 

The Cage group had 116 cases with a mean age of 45.0 (range 27-58). Fifty-five were male, while the remaining 61 cases were female. The most common vertebral level was C5/6 (53.0%). The next most common level of operation was C4/5, which occurred in 21.37% of this group. All patients were either ASA grade 1 or 2. The Z-P group consisted of 117 cases with a mean age of 46.2 years (range 20-60). Of this group, 58 were male and 59 were female. Most patients had few co-morbidities, with only two patients (1.71%) having an ASA grade of 3 and the remainder having either an ASA 1 or 2. The ASA 3 patients were so due to a diagnosis of hypertension with symptomatic coronary artery disease. Similar to the Cage group, the most common vertebral level of operation was C5/6, with a total of 59 patients (50.43%). This was followed by the C6/7 vertebral level (27.35%). There was no significant difference between the two population groups, as shown by the p-values in Table 1 (p>0.05). The mean follow-up for the Z-P and Cage groups was 8.2 months and 7.2 months, respectively (p=0.146).

**Table 1:** Patient demographics and clinical characteristics

	Z-P	Cage	p-value
Number of cases	117	116	
Sex (male: female)	49.6%: 50.4%	47.4%: 52.6%	0.742
Mean age	46.2±7.79	45.0±7.71	0.218
Vertebral level			
C3/4	5.13%	5.98%	0.770
C4/5	16.24%	21.37%	0.351
C5/6	50.43%	52.99%	0.818
C6/7	27.35%	18.80%	0.184
C7/T1	0.85%	0.00%	0.319
ASA grade			
1	11.98%	16.38%	0.371
2	86.32%	82.91%	0.768
3	1.71%	0.00%	0.159
4	0.00%	0.00%	-
Mean follow-up (months)	8.2	7.2	0.146

Inter-observer reliability

The mean ICC for all disc height measurements was 0.79, indicating good reliability. The ICC value for the measurement of s-Cobb was lower, with a value of 0.63, showing a moderate degree of inter-observer agreement. Spondylolisthesis measurements showed excellent inter-observer reliability, with an ICC of 0.91.

Actual disc height (aDH) 

**Figure 5:**
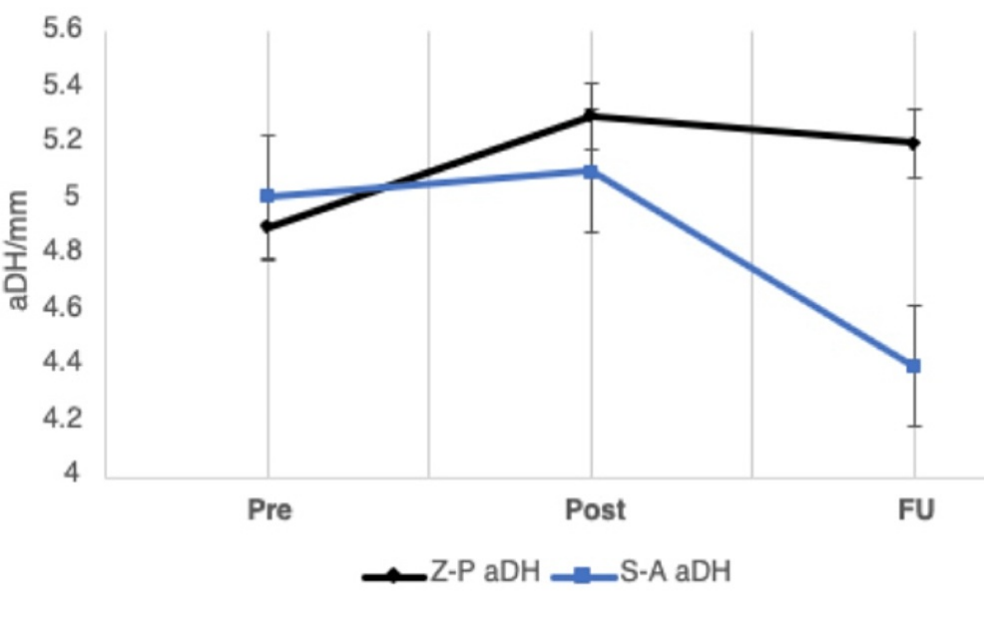
Serial follow-up graph displaying change in aDH for both treatment groups over the three measurement time points (pre, post, and FU) aDH: actual disc height; Pre: pre-operative; Post: post-operative; FU: follow-up

Mean aDH values are displayed in Table 2. There was no significant difference between the mean pre-operative aDH in both groups (p = 0.308). At the follow-up stage, mean aDH values showed improvement in the Z-P group, while aDH was diminished in the Cage group. The aDH increased immediately post-operatively in both groups, with a mean increase in disc height of +0.4±0.94 in the Z-P group and +0.1±1.00 in the Cage group (p=0.001) (Table 2). Compared to initial disc height, this was also observed after the follow-up period in the Z-P group, with a loss of disc height in the Cage group (p=0.007). In both groups, aDH decreased between the post-operative and follow-up stages, as shown in Figure 5. The difference in the post-operative and follow-up aDH was significantly different between both groups, with the Z-P group demonstrating maintained aDH (p<0.001). A total of 63 (27.0%) cases had zero subsidence in the post-operative and follow-up periods. Of this cohort, 48 (76.2%) were treated with the Z-P implant, compared to 15 (23.8%) from the Cage group (p<0.001). Subsidence of >2mm was classified as clinically significant, as this indicates vertebral susceptibility to collapse due to a reduction in disc height. The Z-P group had 0 cases with this degree of collapse in comparison to 6 (5.17%) in the Cage group (p<0.001). Most cases had subsidence of <2mm.

**Table 2:** Change in post-operative actual vertebral disc height (aDH) Pre: pre-operative; PO: post-operative; FU: follow-up

	Z-P	Cage	p-value
Mean ADH / mm			
Pre	4.90±0.83	5.01±0.77	0.308
PO	5.30±0.71	5.10±0.81	0.002
FU	5.20±0.66	4.40±0.95	<0.001
Mean change in ADH / mm			
Pre-PO	+0.4±0.94	+0.1±1.00	0.001
Pre-FU	+0.3±0.92	-0.6±1.09	0.007
PO-FU	-0.2±0.35	-0.7±0.67	<0.001
Subsidence			
0mm	48 (41.0%)	15 (12.9%)	<0.001
<2mm	69 (59.0%)	95 (81.9%)	0.037
³2mm	0 (0%)	5 (4.31%)	0.025
³3mm	0 (0%)	1 (0.86%)	0.315

Segmental Cobb angle (S-Cobb)

**Figure 6:**
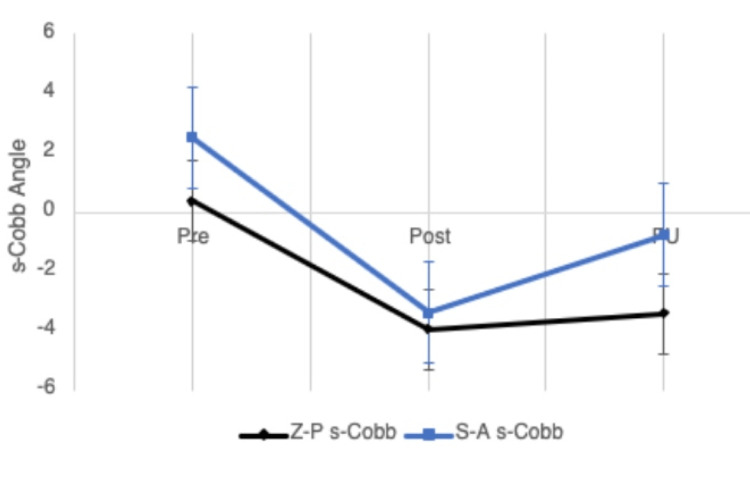
A serial follow-up graph displaying the change in s-Cobb angle for both treatment groups over the three measurement time points (pre, post, and FU). Above the horizontal line at point 0 indicates a kyphotic angle, while below this line indicates a lordotic angle. s-Cobb: segmental Cobb angle; Pre: pre-operative; Post: post-operative; FU: follow-up

Both implants achieved the restoration of cervical lordosis immediately after surgical intervention (Figure 6). The mean difference in s-Cobb from pre-operative to post-operative was -5.9°±6.0 for the Cage group and -4.0°±5.7 in the Z-P group (p=0.016). These angles gradually became more kyphotic between the post-operative and follow-up reviews.

Compared to the post-operative measurements, there was a recurrence of kyphosis in both groups, as s-Cobb kyphosed by 2.6°±6.1 in the Cage group and 0.5°±2.3 in the Z-P cohort (p=0.009) (Table 3). The mean pre-operative to post-operative and pre-operative to follow-up s-Cobb differences were not significantly different between the two groups (p>0.05). The incidence of kyphosis at the FU stage showed a significant difference between the Cage and Z-P groups, with the Z-P group displaying less post-operative kyphosis (p<0.001). Only one case had kyphosis in this group in comparison to 40 cases in the Cage group.

**Table 3:** Change in segmental Cobb angle (s-Cobb) with regard to the pre-operative, post-operative, and follow-up periods Pre: pre-operative; PO: post-operative; FU: follow-up *A negative value indicates a lordotic angle, whilst a positive value indicates a kyphotic angle

	Z-P	CAGE	p-value
Change in S-Cobb (°)*			
Pre-PO	-4.0±5.7	-5.9±6.0	0.016
Pre-FU	-3.5±6.0	-3.3±7.2	0.578
PO-FU	0.5±2.3	2.6±6.1	0.009
Kyphosis incidence at FU (%)	1 (0.85%)	40 (34.48%)	<0.001

Spondylolisthesis 

**Figure 7:**
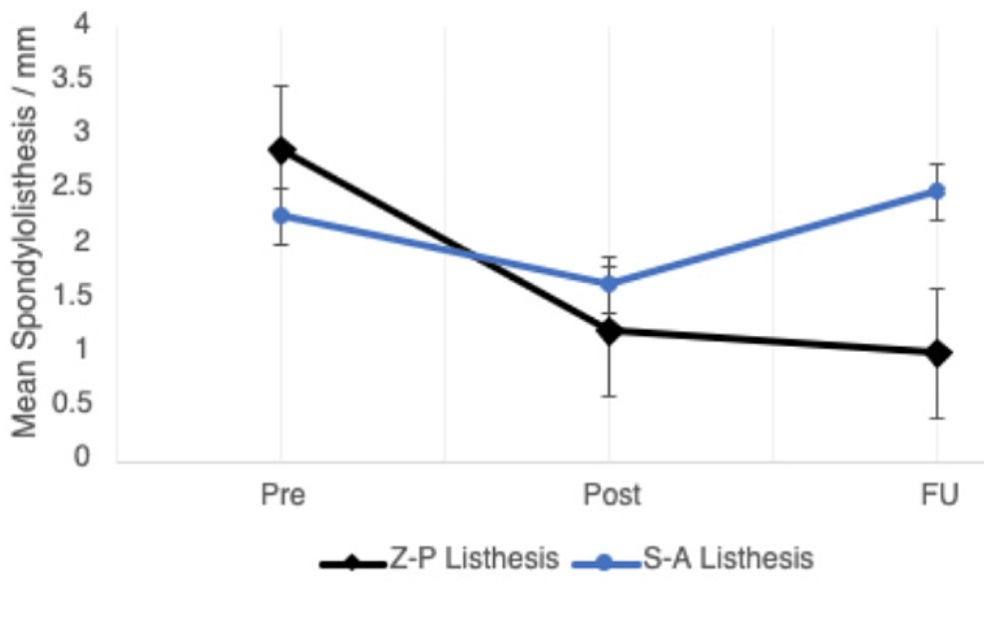
A serial follow-up graph displaying change in spondylolisthesis for both treatment groups over the three measurement time points (pre, post, and FU). Pre: pre-operative; Post: post-operative; FU: follow-up; Listhesis: spondylolisthesis

A total of 44 cases (18.9%) had significant spondylolisthesis pre-operatively, as defined by Tani et al. [13]. Incidence was decreased post-operatively in both groups, with eight cases in the Cage group and one case in the Z-P group having unresolved spondylolisthesis. This number remained the same at follow-up in the Z-P group; however, spondylolisthesis cases increased in the Cage group (Table 4, Figure 7). The mean spondylolisthesis values at the PO and FU stages were significantly different between both groups (p<0.05). There was a significant reduction in spondylolisthesis displacement immediately post-operatively in both groups. The Z-P group demonstrated the greatest change in listhesis with a mean DListhesis of -2.82±1.66 compared to -1.82±1.20 in the Cage group (p<0.001). In the follow-up period, spondylolisthesis recurred in the Cage group, with a mean change in spondylolisthesis of 0.81±1.34. Cervical alignment improved significantly during this period in the Z-P group compared to Cage (p<0.001).

**Table 4:** Change in spondylolisthesis over the pre-operative, post-operative, and follow-up periods Pre: pre-operative; PO: post-operative; FU: follow-up *A negative value indicates a reduction of spondylolisthesis, thus restoring normal cervical alignment. A positive value indicates the recurrence of spondylolisthesis.

	Z-P	Cage	p-value
Spondylolisthesis incidence			
Pre	22 (18.8%)	22 (19.0%)	0.977
PO	1 (0.85%)	8 (6.90%)	0.019
FU	1 (0.85%)	15 (12.9%)	<0.001
Mean spondylolisthesis /mm			
Pre	2.87±1.64	2.26±0.75	0.143
PO	1.20±0.00	1.63±0.58	0.020
FU	1.00±0.00	2.49±0.93	<0.001
Mean change in spondylolisthesis* /mm			
Pre-PO	-2.82±1.66	-1.82±1.20	<0.001
PO-FU	-0.01±0.04	0.81±1.34	<0.001

## Discussion

Surgical management 

Symptomatic cervical disc disease presents with clinical syndromes such as myelopathy and radiculopathy. Failure to manage these symptoms by conservative means leads to the requirement of surgical intervention. The most common procedure performed on these patients is ACDF. This has historically provided successful arthrodesis and restored segmental stability. Since the 1980s, anterior plating has been common practice, improving the fusion rate and maintaining cervical lordosis in both single-level and multi-level cases ​[3,6]. However, plating is not without complications, with multiple studies reporting significant incidences of dysphagia and screw migration ​[7,8]. This has since resulted in the development of interbody cages and Z-P devices. It is for these reasons that anterior plating is not common practice in Northern Ireland, and the two implants in this study are the first choices.

Study demographics 

The demographic differences between the Cage and Z-P groups in this study were insignificant (sex, age, and mean follow-up). The most common level of operation for both groups was C5/6. This goes against the most affected level for cervical spondylosis, which is C6/7 [14]. This is due to the transition from the more mobile cervical spine to the less mobile thoracic spine. The reason behind this is that clinical syndromes caused by insults to the C5/6 level are more pronounced. The resulting impingement of the C6 nerve root causes pain, paraesthesia, and weakness in the thenar region, wrist extension, and elbow flexion. Neck pain also occurs, which alone has a point prevalence of 0.4%-41.5% [15]. All patients with the C5/6 level affected in this study experienced persistent neck pain and a combination of the symptoms listed previously.

Complications and revision 

**Figure 8:**
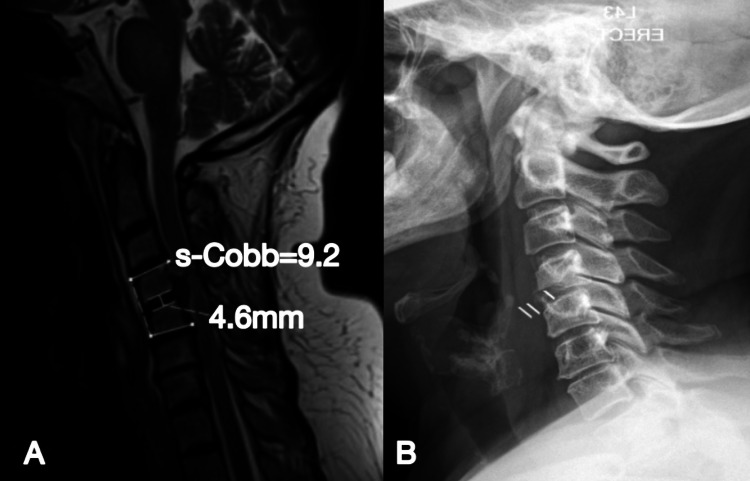
(A) Pre-operative sagittal T2 MRI showing the significant degenerative disc disease prior to treatment with a stand-alone cage; (B) Sagittal x-ray at six months follow-up displaying migration of the Cage and segmental collapse of the C4/5 level

A review of complications following ACDF procedures from 1989 to 2019 revealed a morbidity rate of 13.2%-19.3%. In this figure, the most common complication was post-operative dysphagia. Re-operation rates for these procedures range from 9.13% to 11.1% in single-level cases and 17.6% in multi-level cases [16-18]. The re-operation rate for this study was only required for one case (0.43%). This case was treated initially with the Cage construct and was readmitted six months post-operatively with neck pain and radiculopathy. Radiographic imaging showed segmental collapse, kyphosis, and spondylolisthesis (Figure 8). A primary cause for revision is inadequate arthrodesis, according to the four major fusion criteria. These are trabecular bone bridging between endplates; no radiolucent gap; absence of translation between vertebral bodies on flexion and extension; and absence of motion between spinous processes on flexion and extension [19].

**Figure 9:**
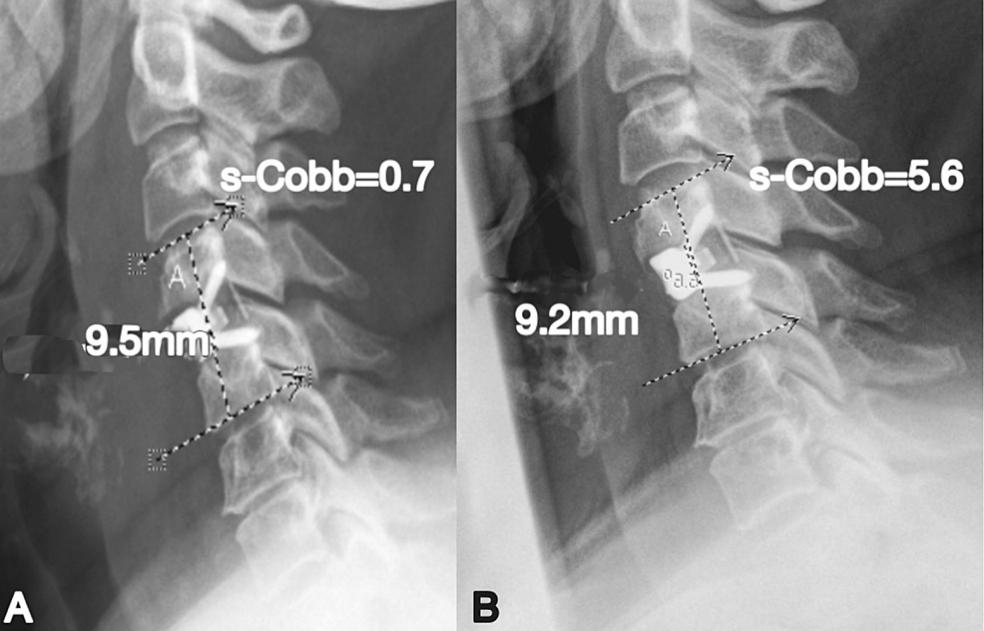
A) Day one post-operative sagittal x-ray displaying Z-P positioning. Disc height = 9.5mm with a lordotic s-Cobb of 0.7 (B) Six-month follow-up showing maintenance of disc height (9.2mm) and lordosis (5.6)

This was followed by a revision of ACDF with the Z-P device. This patient was reviewed at the three-month and six-month follow-up stages. They were asymptomatic at both time points, and radiographs displayed restoration of cervical lordosis in addition to maintenance of disc height (Figure 9).

Change in intervertebral disc height

Both implants were successful in increasing disc height immediately postoperatively; however, the Z-P group had significantly superior results at this stage (p = 0.001). This was also the case at follow-up, with the Cage showing a loss of disc height compared to the day one post-operative measurement (-0.7±0.67mm). The reason behind this is proposed to be due to increased focal stress on the cage, causing a loss of disc height ​[20,21]​. Despite clinical complications being beyond the scope of this study, subsidence >2mm has been associated with increased clinical complications and poor patient-reported outcomes ​[11,22-24]​. Subsidence incidence was relatively high in both groups; however, it was significantly higher in the Cage group compared to the Z-P group (p<0.001). Savio et al. [13] reported a mean subsidence incidence of 28.9% for Cages in their systematic review. It has also been reported that most subsidence cases are clinically significant, with 63% between 2 and 4mm and 9% >5mm [2]. These incidences are significantly greater than those found for both single and multi-level cases in the Cage group in the Northern Irish cohort in this study (5.17%). There is also a direct association between subsidence and loss of cervical lordosis, as subsidence incidence increases in patients with cervical malalignment [21].

Restoration of cervical lordosis 

Segmental Cobb angles were also evaluated, with both groups displaying restoration of cervical lordosis post-operatively. At follow-up, this lordosis reduced in both groups as time progressed; however, the Cage group demonstrated a significantly increased incidence of post-operative kyphosis (p<0.001). Segmental kyphosis has been linked to Cage use, with a mean loss in lordosis of 1.84° observed by Toni et al. [13]. In this unit, a mean kyphotic change of 2.6° was observed post-operatively in the Cage group. Furthermore, 76.8% of cases with a clinically significant kyphotic change (>3°) were treated with the Cage implant. These results further support the pre-existing literature, associating PEEK stand-alone cage constructs with post-operative kyphosis [4, 9]. The reasoning behind this can be due to a multitude of factors; however, as the Cage group was more prone to subsidence, this caused increased axial loading of the anterior vertebral column. This in turn leads to vertebral body wedging and, subsequently, segmental kyphosis [22]. 

Spondylolisthesis 

Degenerative cervical spondylolisthesis is not uncommon and has received an inadequate amount of attention. In this study alone, 18.9% had significant spondylolisthesis >2mm. Very few studies have measured the outcomes of ACDF procedures in degenerative spondylolisthesis patients. This current study measured it at each time point to compare the effectiveness of each implant in treating this condition. The Z-P implant was effective in both reducing the number of spondylolisthesis cases and their magnitude. However, this was not the case in the Cage group, as there was a recurrence of this condition in 68.2% of those who had it pre-operatively. This is likely due to the absence of a fixation mechanism in the Cage, therefore allowing anterior translation after repetitive flexion, extension, and axial loading ​[25,26]​. Furthermore, as ACDF procedures involve the removal of the annulus fibrosus and posterior longitudinal ligament, anatomical resistance against spondylolisthesis is lost [27].

Implant choice 

Evidence has demonstrated the benefit of using Z-P in comparison to conventional anterior plating due to the fixed-angle augmented screws providing a more rigid construct ​[8,9]. Furthermore, the procedure for Z-P implantation has a reduced operation time and decreased dysphagia incidence post-operatively compared to plating [28]. The reasoning behind this is that inserting the Z-P device reduces oesophageal stimulation and thus reduces the risk of oesophageal oedema and adhesions ​[29,30]. As anterior plating is not routinely performed in the spinal centre in Northern Ireland, it would be unjust to comment on our experience with this particular procedure and compare it against Z-P or Cage implants. A study by Cho et al. [9] reported that the Z-P implant had superior radiological outcomes compared to Cages. Disc height, subsidence, and Cobb angles were analysed; however, spondylolisthesis severity was not documented. In the current study, similar parameters were measured to those mentioned above, finding the same results.

Clinical outcomes 

The main limitations of this study are, firstly, its retrospective nature, and secondly, the fact that clinical outcomes were not assessed. Despite the association between radiographic and clinical outcomes, it is not possible to draw that conclusion in this study. As there is an economic difference between the two devices, it would be worth correlating these results with the cost-effectiveness of using the Z-P implant. This is of particular interest as the majority of ACDF procedures performed in Northern Ireland are covered by the National Health Service (NHS).

## Conclusions

Overall, the regional spinal surgery centre in Northern Ireland produces satisfactory radiographic results for ACDF procedures. Compared to the Cage without screw augmentation, the Z-P augmented screw device proved more successful at reducing subsidence, maintaining cervical lordosis, and treating degenerative spondylolisthesis. These results are based entirely on radiographic outcomes. Clinical outcomes for patients treated with both implants need to be correlated with the radiographic results. Further research is required in the comparison between these two implants. A study combining an extended follow-up and the inclusion of clinical parameters would provide data that would aid clinical decision-making. This study, however, with consideration of patient characteristics, advocates a cautious endorsement for the use of the Z-P implant.
